# Alarm fatigue and moral distress in ICU nurses in COVID-19 pandemic

**DOI:** 10.1186/s12912-022-00909-y

**Published:** 2022-05-24

**Authors:** Neda Asadi, Fatemeh Salmani, Narges Asgari, Mahin Salmani

**Affiliations:** 1 Nursing Research Center, Kerman University of Medical Sciences, Kerman, Iran; 2 Nursing and Midwifery Sciences Development Research Center, Najafabad Branch, Islamic Azad University, Najafabad, Iran; 3 9 DAY Manzariyeh Hospital, Isfahan University of Medical Sciences, Isfahan, Iran; 4 Department of Mathematics and Statistics, University of New Brunswick, Fredericton, Canada

**Keywords:** COVID-19, Fatigue, Moral distress, ICU, Nurse

## Abstract

**Introduction:**

Most ICU nurses feel overwhelmed by the variety of alarms at the same time. Therefore, nurses experience very stressful situations in relation to many responsibilities and care demands. This stressful condition has recently been exacerbated by COVID-19 and potentially endangers patient safety. The aim of this study was to investigate the alarm fatigue and moral distress of ICU nurses in COVID-19 crisis.

**Method:**

This is a descriptive-analytical cross-sectional study (April-May 2021). Sampling was done by convenience among ICU nurses affiliated to Isfahan University of Medical Sciences, Iran. Data were collected using Nurses’ alarm fatigue and the moral distress scale (MDS). Data were analyzed using ANOVA, independent t-test and multivariate logistic regression.

**Result:**

The results showed that the mean score of alarm fatigue was moderate)19.08 ± 6.26 (and moral distress was low (33.80 ± 11.60). The results showed that there was a significant relationship between alarm fatigue and related training courses)*P* = .012(.So that, alarm fatigue in nurses who were trained in working with ventilators and alarm settings was significantly less than other nurses. Also, a significant relationship was found between moral distress and marital status(*P* = .001) and Shift type(*P* = .01). On the other hand, the risk of alarm fatigue was higher in participants who have a PhD. The results showed that no significant correlation was found between alarm fatigue and moral distress (*r* = 0.111, *P* = 0.195).

**Conclusion:**

It is suggested that practical training courses on alarm management be included in the curriculum and the ICU nurses should have practical training before starting work in the ICU and on an annual basis. In order to protect nurses and ensure quality care of patients, nurse managers should reduce the number of rotating shifts of ICU nurses.

## Introduction

Intensive care unit (ICU) nurses provide the highest level of care to critically ill patients. They have to expend a high amount of energy to provide care and meet the critically ill patient’s needs [1–3]. Health care systems around the world have been overwhelmed by the rapid spread of COVID-19 [4–6]. High workload, long-term fatigue, the threat of COVID-19 disease and frustration resulting in the death of nurses who care for them are all among these factors. If these problems are not solved, especially nurses’ fatigue, the quality and safe-care of patients will also be affected [7–9].

One of the most effective factors that can cause fatigue in ICU nurses are electronic equipment and monitors and intensive care devices. With the advancement of technology, monitoring devices that have alarm and warning systems are widely used in ICU departments. With the sound of device alarms, there is a need for nurses to respond appropriately to improve the situation based on the patient’s needs [10].

Most nurses feel overwhelmed by the number and variety of alarms that sound at the same time [11]. False alarms or unusual sounds made by multiple devices are known to be a source of stress for ICU nurses [12]. Desensitization to these alarms is defined as alarm fatigue [13]. According to the results of the Simpson 2019 study, most of the generated alarms are not related to the patient’s clinical condition and despite the immediate response of the nurses, did not help the patient’s clinical evaluation and caused fatigue in the nurses [14], a condition that has recently been exacerbated by COVID-19 and potentially endangers patient safety [15].

Therefore, nurses experience stressful situations in relation to many responsibilities and care demands [16]. Moral distress is one of the critical problems in the workplace that is very neglected and can limit the performance of nurses to provide quality care [17]. In fact, it causes moral dysfunction despite having the necessary knowledge [18, 19]. Moral distress is the feeling of unhappiness that occurs despite not recognizing the proper moral action. Nurses’ exposure to moral distress can lead to different outcomes for nurses and patients and affect treatment outcomes. In addition, moral distress in nurses can cause symptoms such as sadness, frustration and anxiety, and in the long-term can lead to burnout and job dissatisfaction [20–22]. The results of studies in this field also show that moral distress has a significant relationship with the service sector and the highest level of moral distress is related to the ICU [19].

ICU nurses respond in a variety of ways to morally stressful situations. Some nurses, despite their desire, have to endure the existing conditions, preferring to refrain from attending the patient’s bedside and providing care to them, and in fact, they experience a kind of frustration and fatigue in providing care to patients [20, 23, 24]. As the COVID-19 epidemic continues, ICU nurses are at the frontline of the fight against the disease. In this case, monitoring alarms are an important principle in patients care. The use of a large number of electrical devices as well as the combination of alarms has caused alarm fatigue in nurses, which can be dangerous for patients and affect patient safety, as this is one of the 10 major technological threats in health systems [10]. On the other hand, due to the nature of the ICU and the high prevalence of moral distress in these nurses [21], especially during the COVID-19 pandemic, it is important to pay attention to these issues, because it can affect the quality of patient care. Since the researcher did not find a similar study in this regard, the current study conducted with the question of how much is the alarm fatigue and moral distress in ICU nurses during the COVID-19 pandemic This study aimed to determine the correlation between ICU nurses’ background characteristics and their moral distress and alarm fatigue in Iran.

## Methods

### Sample and setting

This is a descriptive-analytical cross-sectional study (April-May 2021). Sampling was done by convenience among ICU nurses affiliated to Isfahan University of Medical Sciences, Iran. Inclusion criteria included: There is no hearing disorder and no history of definite and proven psychological disorder based on self-report. Exclusion criteria included completing less than two-thirds of the questionnaire. An online survey was conducted through social media (WhatsApp) and personal contacts. A brief written description of the study and its objectives was sent to participants. one-hundred fifty participants were considered as the sample size according to pilot study and correlation coefficient, 95% confidence, and 90% test power. Finally, 10 participants were excluded from the study.

### Ethics approval and consent to participate

This study was approved by the Ethics Committee of Kerman University of Medical Science with No. 99001118 and the code of ethics No IR.KMU.REC.1399.679. All methods were carried out in accordance with relevant guidelines and regulations. Participation in this study was voluntary. All participants were given explanation aboutthe objectives and process of the study and their informed consent was obtained.

### Measures

Data were collected using a three-part questionnaire consisted of demographic information, Nurses’ alarm fatigue questionnaire, and Distress Moral Scale (MDS).

Background information questionnaire, included demographic information such as age, gender, marital status, educational level.) and information about occupational conditions (Shift type, Number of night shifts, Second Job, Sleep Disorder, Ventilator training experience, Work experience in the intensive care unit).

#### Nurses’ alarm fatigue questionnaire

It was developed by Torabizadeh in 2017. “Alarm sounds make me nervous” and “I turn off the alarms at the beginning of every shift” are example items this measure. This scale consisted of 13 items and scored based on a 5-point Likert scale (0 = never) t0(4 = always), Two items of this questionnaire have a negative score (items 1 and 9), so the total score of the scale is between 8 and 44. The highest score indicates the highest level of Nurses’ alarm fatigue. The test of the reliability of this questionnaire based on the internal homogeneity and retest methods yielded the following results: test–retest correlation coefficient = 0.99; Guttman splithalf correlation coefficient = 0.79; Cronbach’s alpha = 0.91 [25]. In the current study, the content validity index was determined to be 0.89. Also, the reliability (internal consistency) of this questionnaire was confirmed with a Cronbach’s alpha coefficient of 0.91.

#### Distress moral scale (MDS)

MDS was designed to assess moral distress in nurses working in ICU and CCU and has been used in many studies. Content Validity Index was found to be 0.97 for moral sensitivity and 0.88 for moral distress questionnaires. Intraclass Correlation Coefficient was used to ensure reliability. To this end, questionnaires were completed by 15 participants twice with a 2-week interval. Reliability was found to be 0.93 for moral sensitivity questionnaire and 0.88 for moral distress questionnaire. To determine internal consistency of the tools, Cronbach’s alpha coefficients were found to be 0.77 for moral sensitivity and 0.87 for moral distress [26].

MDS contains 24 items, each relating to a particular situation in hospital care.“When a patient’s death is inevitable, I talk to her family about organ donation” and “I do not react if I see cases where the patient’s privacy is violated.” are example items this measure. This scale examines nurses’s understanding of two aspects: “severity of moral distress” and “frequency of stressful situations faced by the individual.” In this scale, the nurses specify how frequently they face stressful situations that may cause moral distress in them. It also assesses how intense each of these stressful situations is. This tool has two 5-point Likert scales: one for frequency of moral distress (from never = 0 to very much = 4) and the other for severity of moral distress (from causing no distress = 0 to causing severe distress = 4), with score range from 0 to 96. Higher scores indicate higher intensity and frequency of moral distress.

SPSS 25 was used for data analysis. Frequency, percentage, mean and standard deviation were used to describe the demographic characteristics and the scores of nurses’ alarm fatigue and MDS in participants. To determine the relationship between nurses’ alarm fatigue and MDS with background variables was used of ANOVA and independent t-test. The significance level was considered 0.05. The logistic regression was conducted for further analysis.

## Results

The mean age of the study participants was 35.1 ± 7.43 years. The majority of the participants were women (96.4%), married (77.9%), had a Bachelor’s degree (75%),worked on rotation shift (81.4%), and had ventilator device management training (70%). The mean Work experience in the intensive care unit of the study participants was 6.91 ± 5.22 years. Other participants’ characteristics are presented in Table 1.

**Table 1:** Demographic characteristics of the participants

Group	Variable	Frequency(percent)	Alarm fatigue	statistic test *P* value	Moral distress	Statistic test *P* value
Gender	Women	135 (96.4)	19.22 ± 6.30	t = 1.41 *P* = .159	33.37 ± 11.81	t = −0.353 *P* = .725
	Men	5 (3.6)	15.20 ± 3.56		35.60 ± 0.54	
Marital status	Married	109 (77.9)	19.01 ± 6.11	t = .245 *P* = .807	32.07 ± 11.45	t = 3.39 P = .001
	Unmarried	31 (22.1)	19.32 ± 6.86		39.81 ± 10.15	
Educational level	Bachelor	105 (75)	18.76 ± 6.32	F = 1.98 *P* = .141	33.57 ± 12.14	F = .116 *P* = .89
	Master’s	30 (21.4)	19.30 ± 5.63		34.70 ± 10.37	
	PhD	5 (3.6)	24.40 ± 7.40		33.20 ± 7.36	
Shift type	Rotation shift	114 (81.4)	19.55 ± 5.89	t = −1.89 *P* = .06	35.01 ± 11.02	t = − 2.61 *P* = .01
	Fixed Shift	26 (18.6)	17 ± 7.44		28.54 ± 12.75	
Number of night shifts	0	20 (14.3)	19.15 ± 5.77	F = 1.31 *P* = .192	32.40 ± 15.77	F = 1.15 *P* = .315
	1-3	8 (5.7)	18.25 ± 7.25		33.17 ± 14.78	
	4-6	39 (27.9)	17.94 ± 7.07		33.23 ± 10.37	
	7-9	41 (29.3)	19.10 ± 6.10		32.95 ± 1.47	
	> 10	32 (22.9)	21.08 ± 4.40		34.96 ± 9.75	
Second Job	Yes	10 (7.1)	18.90 ± 6.29	t = −.093 *P* = .926	35.89 ± 1.05	t = .558 *P* = .578
	No	130 (92.9)	19.09 ± 6.28		33.65 ± 11.98	
Sleep Disorder	Yes	40 (28.6)	19.23 ± 6.16	t = −.174 *P* = .862	34.15 ± 12.26	t = .225 *P* = .823
	No	100 (71.4)	19.02 ± 6.32		33.66 ± 11.39	
Ventilator training experience	Yes	98 (70)	18.21 ± 6.70	t = −2.54 P = .012	32.85 ± 11.46	t = −1.47 *P* = .142
	No	42 (30)	21.10 ± 4.53		36 ± 11.74	

The mean score of alarm fatigue was 19.08 ± 6.26. So that the ventilator device of alarm fatigue in nurses is moderate. The mean score of moral distress was 33.80 ± 11.60. So that the moral distress in nurses is low. No significant correlation was found between alarm fatigue and moral distress (r = 0.111, *P* = 0.195).

The multivariate logistic regression with enter method was conducted for further analysis. The results showed that gender and level of education were significantly associated with alarm fatigue. On the other hand, the risk of alarm fatigue was higher in women and participants who have a PhD. However the coefficient of adjusted R square (R2) of the final model was 0.072, the two variables of gender and level of education significantly explain 7.2% of the changes in alarm fatigue scores. (Table 2).

**Table 2:** The logistic model of associations of important variables with alarm fatigue

Variable	Multivariate logistic regression			
	Unstandardized β	standardized Coefficients β	t	*P* value
Age	−.091	−.108	−.830	.408
Gender	−8.642	−.257	−2.135	.035
Marital status	1.618	.108	1.173	.243
Educational level	2.528	.213	2.235	.027
Shift type	3.443	−.176	2.167	.132
Number of night shifts	−.165	−.158	−1.851	.066
Second Job	−4.256	−.176	−1.514	.132
Sleep Disorder	−.245	−.018	−.207	.835
Ventilator training experience	1.898	.139	1.571	.119
Work experience in the intensive care unit	.161	.135	1.08	.282

Also, the multivariate logistic regression showed that marital statues were significantly associated with moral distress. On the other hand, the risk of moral distress was higher in unmarried people However the coefficient of adjusted R square (R2) of the final model was 0.061, the variable of marital statues significantly explain 6.1% of the changes in moral distress scores. (Table 3).

**Table 3:** The logistic model of associations of important variables with moral distress

Variable	Multivariate logistic regression			
	Unstandardized β	standardized Coefficients β	t	*P* value
Age	−.201	−.129	−.979	.33
Gender	3.939	.063	.489	.626
Marital status	−6.327	−.228	−2.457	.015
Educational level	.954	.043	.452	.652
Shift type	4.495	.152	1.517	.132
Number of night shifts	−.169	−.087	−1.015	.312
Second Job	.364	.008	.062	.95
Sleep Disorder	.464	.018	.209	.835
Ventilator training experience	.680	.027	.301	.764
Work experience in the intensive care unit	.034	.015	.120	.904

## Discussion

The aim of this study was to investigate the alarm fatigue and moral distress in ICU nurses during the COVID-19 pandemic in Iran. The results of the present study showed that the mean score of alarm fatigue in ICU nurses was moderate, which is consistent with previous studies [27, 28]. Bourji et al. (2020) found that the rate of alarm fatigue during the COVID-19 pandemic, in ICU nurses was above average [29]. Also in the present study, alarm fatigue in nurses who were trained in working with ventilators and alarm settings was significantly less than other nurses. Our results are consistent with previous studies, such as Jiasi (2020) et al. [30], Bourji et al. (2020) [29] and Sowan (2015) [31]. This suggests that ventilator and alarm settings training helps nurses manage alarms and can reduce alarm fatigue in intensive care unit nurses.

Another finding of this study was that the level of moral distress of nurses in intensive care units was low. In this regard, Miljeteig (2021) who conducted a study on nurses of all clinical wards, reported that the level of moral distress of nurses during the COVID-19 pandemic was low and states that in cases where the nurse works in managerial positions or if they are transferred to psychiatric and addiction departments for work, they experience higher levels of moral distress. That same study also suggests that the supportive mechanisms such as talking to colleagues, family and friends, the attention and support of managers, and prioritization guidelines as the most helpful in reducing nurses’ moral distress [27]. Furthermore, the study of Karagozoglu et al. (2017), which was conducted before the COVID-19 pandemic in Turkey, shows a low level of moral distress [28], while Norman et al. (2021) reported high levels of moral distress in nurses during the COVID-19 pandemic. They cite the reasons for this as worries about COVID-19’s negative impact on the family, fear of infecting others, and work-related worries [29]. Also, in other studies conducted before the COVID-19 pandemic, the level of moral distress in ICU nurses has been reported to be moderate to high [21, 23, 31, 32]. However, Hamric et al. (2011) reported the level of moral distress in the intensive care units as 92.86 and this level is within the minimum average range [20]. The low moral distress of the nurses in this study may be related to the fact that marital status was an important predictor of this issue and more than 70% of our participants were married and experienced less moral distress. Also,this discrepancy in the results may be due to differences in the questionnaires and difference in scoring and questionnaire cut points In the Hamric et al. study, the Revised Moral Distress Questionnaire (MDS-R) was used, which has different scores and scoring methods than the questionnaire used in the present study. Also, differences in the study population can affect the amount of moral distress. Although Ebrahimi and Corely also used a MDS questionnaire in their studies, the population in these two studies is all nurses working in intensive care units. In the Hamric study, the study population was pediatric and adult’s ICU nurses, while in the present study, the intensive care units include ICU and CCU.

The results of the present study showed that marital status is a predictor of moral distress and has an effect on the degree of moral distress so that those who are not married experience more moral distress, In this regard, Fazljoo et al. found that the mean score of moral distress was higher in singles compared to married nurses [33], while the results of the study of Ness et al. (2021) are not in line with the results of the present study [34]. It seems that the existence of cultural differences and family support systems can be impactful. Family usually provide physical, emotional and financial support and play a curial role in seeking mental health for their beloved ones [35]. Family functioning is defined as the efficient problem-solving procedues and strategies used by family members, such as having social and emotional communication, to this end, family members try to coordinate or adapt to life changes by staying together, negotiating, observing the differences, to protect the family system. However, a comprehensive understanding requires attention to cultural and contextual factors [36]. And also findings may indicate that because married nurses have experienced life stress, they are better able to manage psychological stress than single, resulting in less moral stress.

Furthermore, the results of the present study showed that nurses who worked in rotation shifts experienced more moral distress, which is in line with the results of the study of Ebrahimi et al. (2013, 31]. It seems that facing different working conditions and problems in care and treatment provided in non-office shifts, reduced access to physicians and the absence of head nurses and hospital managers to respond to problems in the treatment process can affect the moral distress of nurses in intensive care units.

In the present study, two variables of gender and education level predict alarm fatigue and found that nurses who are women and have higher levels of education are more likely to experience alarm fatigue. Whittaker (2018) in their study also discusses gender as an effective factor in the occurrence of alarm fatigue, stating that alarm fatigue occurs more often in female nurses [37]. Also Bourji et al. (2020) show that the mean alarm fatigue score was higher in males and in nurses with higher levels of education [38]. But Zhao et al. (2021) show that high education are negatively correlated with alarm fatigue [39]. Perhaps the reason for this difference is in the existence of COVID-19 condition and the type and environment of the study.

## Conclusion and recommendations

The evidence from this study indicate that there is a correlation between alarm fatigue and moral distress with background characteristics and also alarm fatigue is moderate and moral distress is low in ICU nurses in Iran. In the advent of emerging diseases such as the COVID-19 pandemic, despite the lack of information and the emergence of treatment problems in the field of this disease, nurses play a key role in caring for these patients and are under psychological and moral pressure. Therefore, in order to protect nurses and ensure quality care of patients, managers should adopt strategies to deal with nurses’ problems, including alarm fatigue and moral distress.

The present findings suggest several course of action in order to devise and implement strategies and policies to curtail alarm fatigue and moral distress of ICU nurses for nurse managers. It is suggested that nursing curriculum of undergraduate and graduate nursing programs should prepare new graduates with the resources to manage alarm fatigue and moral distress. It is suggested that in order to prevent the occurrence of alarm fatigue, the nurses should have practical training in working with devices and monitors and alarm management before starting work in the critical wards and on an annual basis. Nursing managers can reduce the number of rotating shifts of nurses in order to prevent moral distress in nurses.

This study shows the need for further research to determine and investigate the antecedents and consequences of moral distress and alarm fatigue in nurses working in different wards, considering individual characteristics with different cultures, to help improve better coping with situations that cause such problems in the workplace and quality of care.

### Limitation

This study was performed in the COVID-19 pandemic,so generalization of study results should be limited. Participants are only intensive care nurses and generalization of study results should only be done to this group and not to other nurses. And also conventional sampling was performed therefore the study samples may not be representative of the community.

## Data Availability

The data are available upon request to the corresponding author after signing appropriate documents in line with ethical application and the decision of the Ethics Committee.
